# Characterizing genome-wide changes associated with inbreeding tolerance in cranberry: insights into homozygosity and heterozygosity

**DOI:** 10.3389/fpls.2026.1898625

**Published:** 2026-09-03

**Authors:** Derek Wang, Joseph Kawash, James Polashock, Iman Dehzangi

**Affiliations:** 1 Center for Computational and Integrative Biology, Rutgers University, Camden, NJ, United States; 2 Genetic Improvement of Fruit and Vegetables Laboratory, USDA-Agricultural Research Service, Chatsworth, NJ, United States; 3 Department of Computer Science, Rutgers University, Camden, NJ, United States; 4 Rutgers Cancer Institute, Rutgers University, New Brunswick, NJ, United States

**Keywords:** cranberry breeding, genotyping by sequencing, heterozygosity, homozygosity, inbreeding tolerance, sequencing data

## Abstract

The American cranberry (*Vaccinium macrocarpon*) is an important native North American fruit. Cranberry is a self-fertile woody perennial. As part of an ongoing breeding and genetics program, inbred lines have been developed to help understand the effects of inbreeding in cranberries and to develop near-homozygous lines. Self-pollination can lead to a phenomenon known as inbreeding depression. As the effects propagate through generations, the loss of genetic diversity in offspring can cause reduced vigor, size of fruit and vegetative organs, and overall fitness. Our early observations suggested that different cranberry cultivars might vary in their expression of inbreeding depression. Here, we investigate genomic and phenotypic patterns potentially associated with inbreeding tolerance across multiple selfing cycles, or generations, of four cranberry cultivars: Ben Lear, Stevens, Wilcox, and Pilgrim, which we generated over a period of 27 years. Using genotyping by sequencing data from individuals across each generation, we assessed the offspring for genetic heterogeneity and observed phenotypic signs of inbreeding depression. We quantified contiguous runs of heterozygosity (ROHet) and homozygosity (ROHom) throughout the cranberry genome and show that from early generations, heterozygosity decreases, and homozygosity increases rapidly, correlating with an increase in genomic inbreeding levels. However, the cultivars were remarkably tolerant of inbreeding from a phenotypic standpoint. We also identified candidate genes in regions of maintained heterozygosity, such as those functioning in metabolism and stress responses, whose potential role in inbreeding tolerance warrants further functional validation. Notably, we identified a conserved ROHet in chromosome 11 of Ben Lear containing eight genes. Altogether, this is the first study to present a public repository of cranberry sequencing data from multiple cultivar generations and identification of candidate genes based on ROHet and ROHom trends across consecutive inbred generations of *V. macrocarpon*.

## 1 Introduction

*Vaccinium macrocarpon*, the American cranberry, is a member of the Ericaceae family known for its many health benefits (Kawash et al., 2022). The United States and Canada are the two largest producers of cranberries and dominate the international cranberry market. In the U.S., only a few states produce the majority of cranberries, including Wisconsin, Massachusetts, New Jersey, and Oregon. These states provide favorable conditions for cranberry production, such as acidic soils, low winter temperatures, and an abundant supply of freshwater (Cranberry Production by State 2025, 2025). Although sold fresh in the fall, the bulk of cranberries produced are used for sweetened dried cranberries and juice products (Kawash et al., 2022).

Cranberry is a high-value specialty crop with an estimated market value of US$2.991 billion (Knowledge Sourcing Intelligence LLP, 2024). Although yields have increased in the last decade, due, in part, to the introduction of new cultivars (Diaz-Garcia et al., 2020), there remains the need for continued breeding efforts to improve disease resistance, abiotic stress resilience, and other horticultural traits (Gallardo et al., 2018). The emergence of next-generation genomic sequencing in the last few decades, along with the push to increase crop quality and production, has revolutionized development in the plant genomics field (Marks et al., 2021). This area of study enables the characterization of important genes and alleles that can improve key crop traits such as growth, yield, environmental adaptation, and resistance to pests and disease. There is an abundance of sequencing data for many plant species, including the popular model *Arabidopsis thaliana* and crops such as maize and rice (Marks et al., 2021). However, sequencing data for cranberries is more limited. To date, two chromosome-scale reference quality genomes have been published (Diaz-Garcia et al., 2021; Kawash et al., 2022). One of these was assembled using a 5th-generation inbred self of a common parental cultivar, Ben Lear (Kawash et al., 2022). The inbred line was used to reduce heterozygosity, thereby simplifying genome assembly.

In the field of plant breeding, understanding crop genetics and which genes contribute to the expression of important traits are critical to developing improved crop varieties. Maintaining the crop’s genetic diversity ensures the capacity for important traits such as adaptation, resilience, disease resistance, and sustainability (Joshi et al., 2023; Salgotra and Chauhan, 2023). A relevant and growing concern is the decreasing diversity of berry crops (Debnath, 2016). This can occur when a narrow genetic base is exploited for breeding or when diversity in natural populations is low, such as in cranberries (Rodríguez-Bonilla et al., 2019). Consequently, both wild and cultivated cranberry germplasm may contain limited allelic variation for agriculturally important traits, reducing the genetic resources available for future crop improvement. In the past 50 years, cranberry breeding programs have released cultivars with improved disease resistance, fruit quality, and yield (Kawash et al., 2022). However, the continued use of a relatively narrow genetic base may increase the risk of inbreeding, and cranberries, like several other crop and plant species, can experience inbreeding depression (Bruederle et al., 1996; Charlesworth and Willis, 2009). This highlights the need to better characterize the genomic consequences of inbreeding and identify sources of useful genetic diversity. Previous studies have demonstrated that the effects of inbreeding depression can vary across genotypes and expressed phenotypic traits. The potential effects of inbreeding, largely due to increased homozygosity, increase the likelihood of offspring inheriting two copies of recessive deleterious alleles, leading to reduced fitness as demonstrated in several species, including *Arabidopsis arenosa (*Barragan et al., 2024), maize (Burton et al., 1978), and cassava (Rojas et al., 2009). However, from a breeding standpoint, inbred lines can be crossed to create offspring that exhibit hybrid vigor or heterosis. Heterosis has successfully been used in many crop plants, such as corn, rice, and wheat, for the improvement of yield and other desirable traits (Miyaji and Fujimoto, 2018). Demonstration and utility of hybrid vigor in fruit and vegetable crops include tomato, pepper, melons, cucumber, and many others (Whaley, 1952; Lippert and Hall, 1972; Silva et al., 2021; Singh et al., 2023).

Throughout a period of 27 years, selfed lines were generated from four parental cranberry cultivars: Stevens, Wilcox, Pilgrim, and Ben Lear, which are presented in this study. These lines provide a resource to investigate how repeated selfing alters genome-wide patterns of heterozygosity and homozygosity and whether these genomic changes are associated with phenotypic evidence of inbreeding depression. Specifically, this study addresses two questions: (1) how do genomic patterns of heterozygosity and homozygosity change across successive generations of selfing; (2) is repeated selfing accompanied by changes in horticulturally important traits, including leaf size, fruit size, and seed characteristics? We hypothesized that successive selfing generations would exhibit progressively increasing homozygosity and decreasing heterozygosity, although the magnitude of these changes and their phenotypic consequences might differ among cultivars due to their distinct genetic backgrounds.

To address these questions, genotypic data for each cultivar were generated for up to 6–10 generations using genotyping-by-sequencing (GBS) (Elshire et al., 2011). We quantified contiguous homozygous and heterozygous genomic segments, referred to as runs of homozygosity (ROHom) and runs of heterozygosity (ROHet), respectively, to characterize genome-wide changes associated with inbreeding. In particular, higher ROHom levels in the genome can provide estimates of higher inbreeding levels (Curik et al., 2014). Although these approaches have been applied in several plant species (Barragan et al., 2024), they have not previously been systematically quantified in cranberries. By integrating ROHom and ROHet analyses with phenotypic measurements, this study establishes a genomic framework for understanding the consequences of inbreeding in cranberries and provides foundational knowledge to support future breeding efforts.

## 2 Results

### 2.1 Effects of inbreeding on cranberry plant morphology

**Figure 1:**
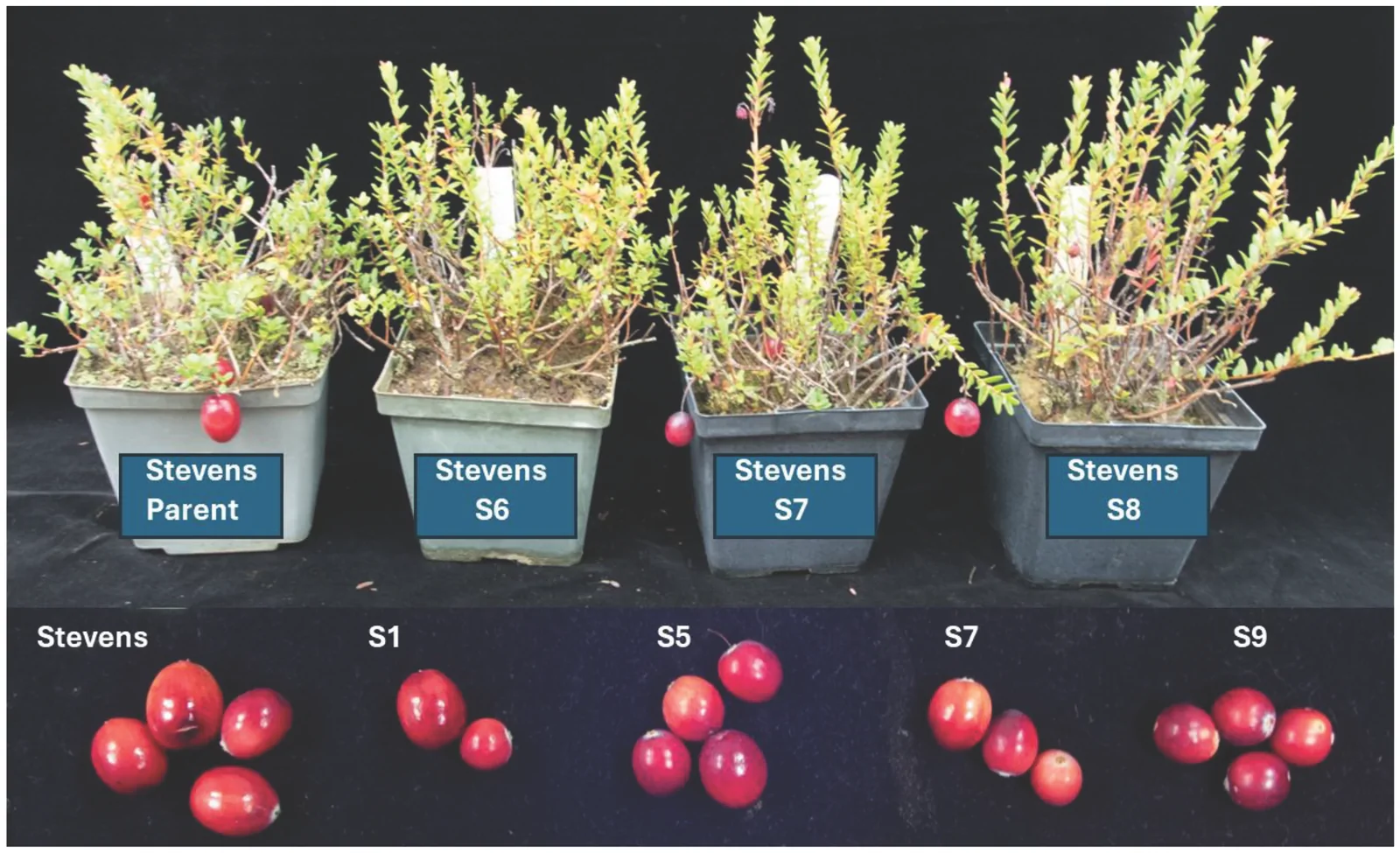
Representative examples of plants and fruit from the ‘Stevens’ inbred line. The inbred generation is indicated by S(N).

Several generations of selfed plants from four cranberry cultivars, Ben Lear, Stevens, Wilcox, and Pilgrim, were phenotyped for signs of inbreeding depression. Representative examples of potted plants were shown for selected generations of the ‘Stevens’ line (**Figure 1**, top). Side-by-side photographs of all cultivars (other cultivars not shown) suggested the leaf sizes and overall plant stature were similar to the parent cultivars, regardless of the level of inbreeding. When the four cultivars were aggregated, leaf area measurements showed some variation among the cultivars (p=0.0525). However, there were no trends based on level of inbreeding (**Supplementary Figure S1A**). Similarly, measurements of fruit size (**Supplementary Figure S1B**) and seed number (**Supplementary Figure S1C**) showed no trends associated with the levels of inbreeding (p=0.46 and p=0.77, respectively). Using one-way ANOVA followed by Tukey’s HSD tests, there were no significant differences detected among generations for leaf size, fruit size, and seed count. Whole fruit appeared slightly smaller than the parent cultivar, but differences were not significant (**Figure 1**, bottom; **Supplementary Figure S1B**).

**Figure 2:**
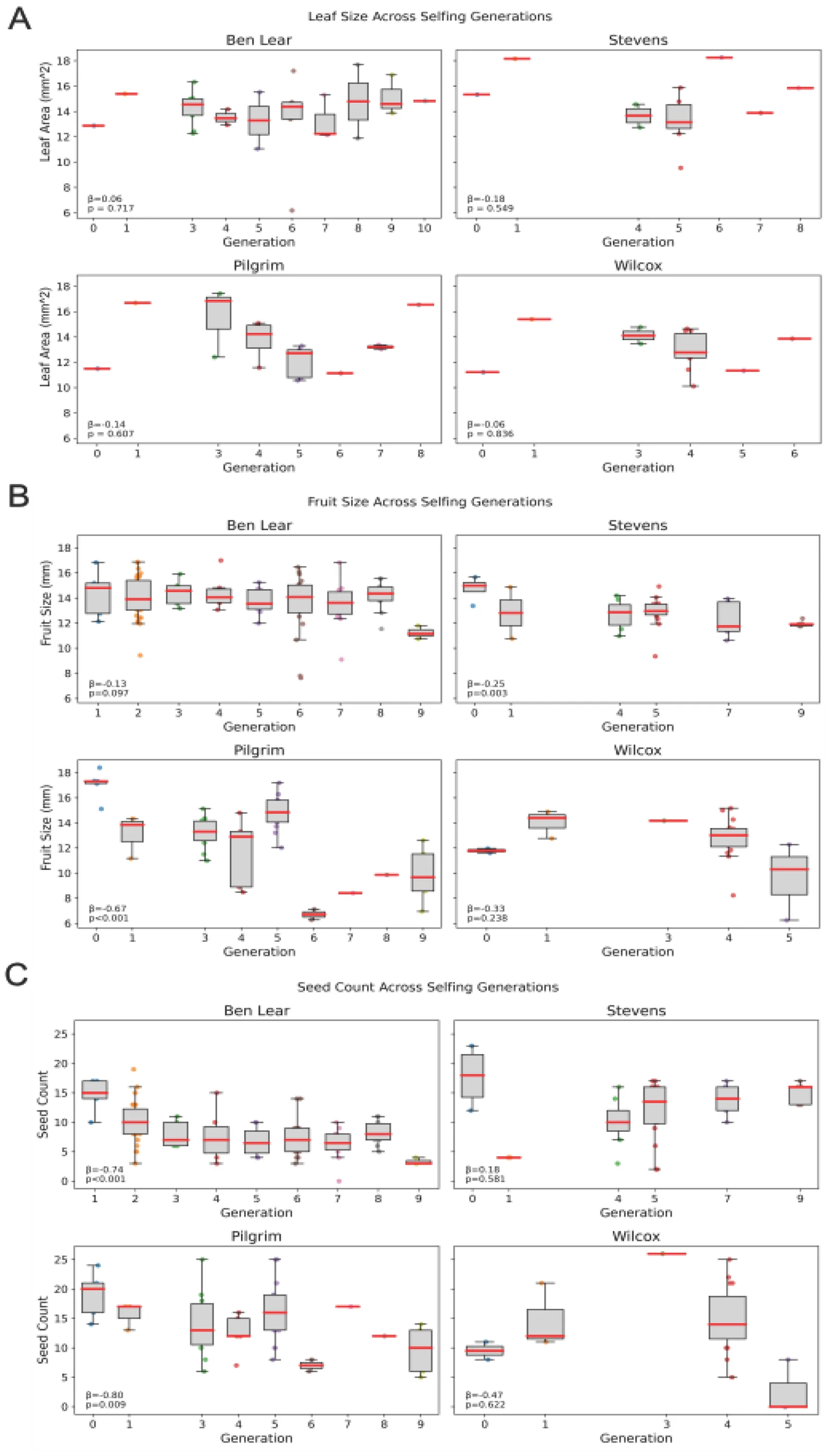
Cultivar-specific distributions of phenotypic traits across consecutive selfing generations. Faceted box plots showing **(A)** leaf size, **(B)** fruit size, and **(C)** seed count for the four cranberry cultivars (‘Ben Lear’, ‘Stevens’, ‘Pilgrim’, and ‘Wilcox’). Each point represents an individual plant measurement. Boxes represent the interquartile range (25th-75th percentiles), the horizontal red line indicates the median, and the whiskers extend to the most extreme observations within 1.5 times the interquartile range. The regression slope (β) and p-value shown in each panel correspond to a linear regression assessing the direction and significance of the association between selfing generation and the measured trait.

To test for cultivar-specific directional trends, linear regression was performed between selfing generation and each phenotypic trait. For leaf area, no significant association with selfing generation was detected in any cultivar (**Figure 2A**). In contrast, fruit size showed a significant negative relationship with selfing generation in ‘Stevens’ (p=0.003) and ‘Pilgrim’ (p<0.001), indicating a reduction in fruit size across successive generations in these cultivars (**Figure 2B**). Similarly, seed count exhibited significant negative trends in ‘Ben Lear’ (p<0.001) and ‘Pilgrim’ (p=0.009), suggesting a decline in seed production with increasing selfing generations in these lines (**Figure 2C**). Despite these cultivar-specific trends, no uniform pattern of phenotypic decline was observed across all cultivars. Taken together, these results suggest that while certain traits in specific genetic backgrounds may be sensitive to increasing levels of inbreeding, there is no consistent evidence of widespread progressive inbreeding depression across these measured phenotypes.

### 2.2 Genomic dataset and alignment

The GBS data from each sequenced generation were aligned to the current ‘Ben Lear’ cranberry reference genome (Kawash et al., 2022). Whole-genome resequencing of the ‘Ben Lear’ selfs were also aligned. After post-processing of the reads, variants were called and then filtered. The variant statistics and total genotyping rates of each line can be found in **Supplementary Table 1**. Genome-wide SNP density varied across chromosomes in all four cranberry cultivars, with markers distributed relatively evenly along the length of each chromosome. Overall, ‘Ben Lear’, ‘Stevens’, ‘Pilgrim’, and ‘Wilcox’ exhibited similar genome-wide marker distribution patterns (**Supplementary Figure S2**).

### 2.3 Identification of ROHet and ROHom and inbreeding levels

**Figure 3:**
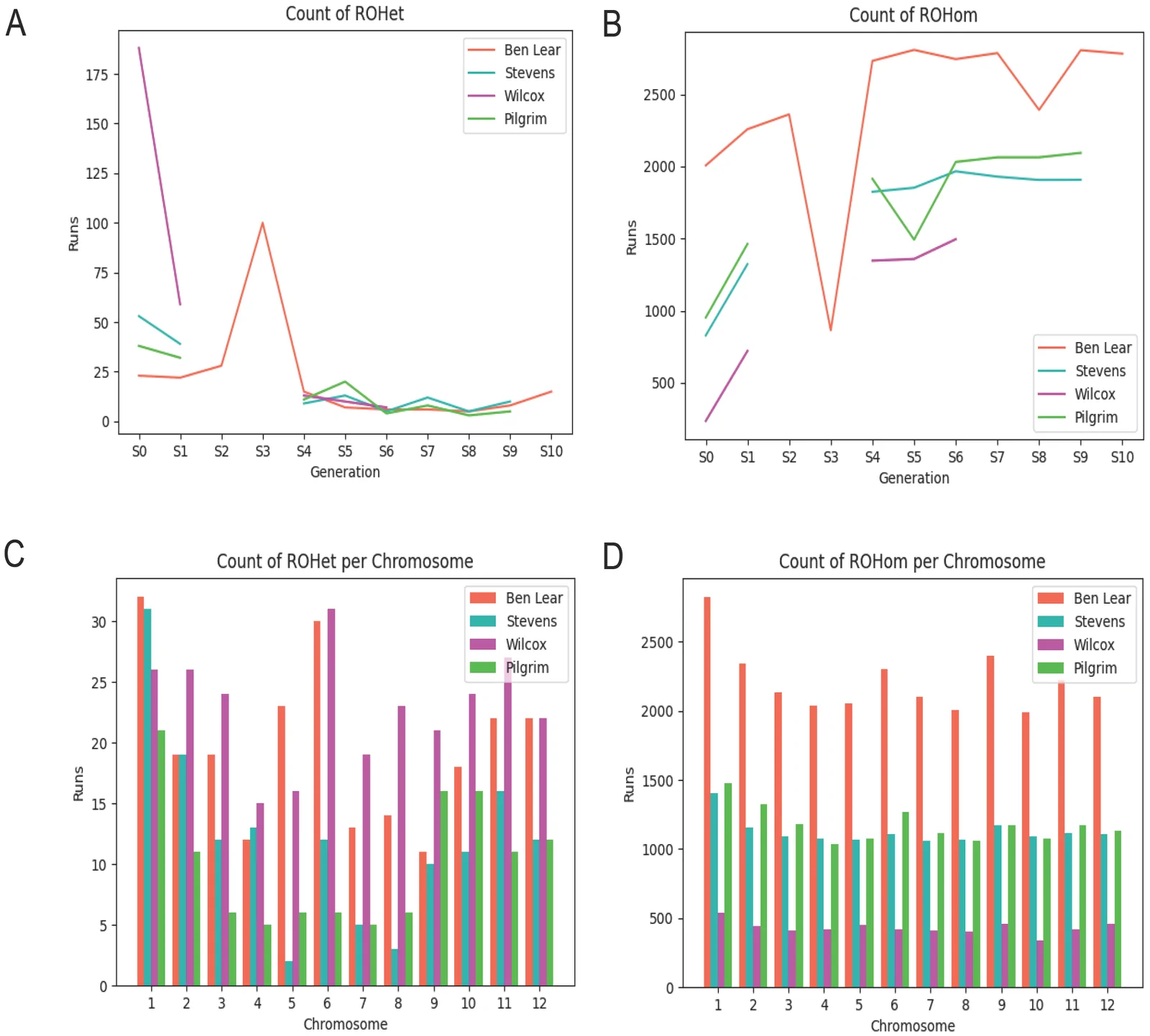
Counts of heterozygous and homozygous runs. **(A)** ROHet counts per generation of the four cranberry cultivars (Ben Lear, Stevens, Wilcox, Pilgrim). **(B)** ROHom counts per generation of the four cultivars. In both graphs, lines are disconnected where sequencing data for that generation is unavailable, either not sequenced (S2 and S3) or due to missing plants. **(C)** ROHet and **(D)** ROHom counts per chromosome of the four cultivars, averaged across all generations.

Using the R package detectRUNS (Detectruns: Vignettes/Detectruns.Vignette.Rmd, 2025), ROHet and ROHom were identified to quantify levels of heterozygosity and homozygosity across generations, respectively. Heterozygosity decreased rapidly in the early generations, yet a minimum level above zero was maintained even by the last generation (**Figure 3A**). This minimum level was similar across the four cultivars. Homozygosity increased quickly during the early generations and leveled out as the generations progressed (**Figure 3B**). A transient increase in ROHet and decrease in ROHom observed in generation 3 of ‘Ben Lear’ (**Figures 3A, B**) were detected using the GBS dataset but were not recapitulated in the corresponding WGS data, suggesting a technical artifact in the GBS data in this generation. To determine whether particular chromosomes exhibited consistently higher or lower numbers of ROHet or ROHom, we calculated the average number of ROHet and ROHom for each chromosome across all available generations within each cultivar (**Figures 3C, D**). However, no chromosomes exhibited significantly higher or lower frequencies of ROHet or ROHom across cultivars, indicating no evidence of chromosomal positional enrichment. ROHet and ROHom counts can be found in **Supplementary Table 2**.

**Figure 4:**
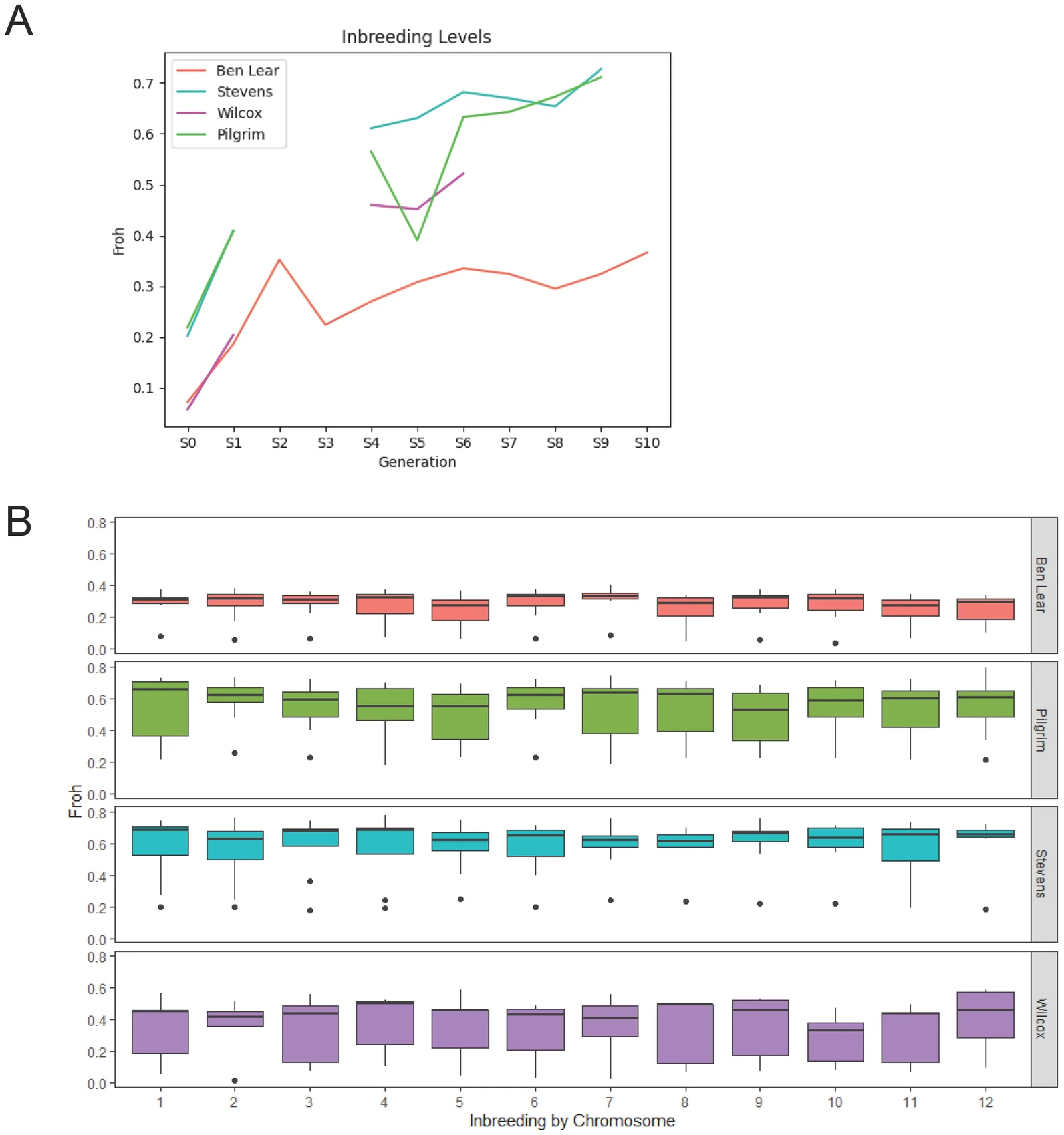
Inbreeding trends across the four cranberry cultivars (Ben Lear, Stevens, Wilcox, Pilgrim). **(A)** Inbreeding levels, represented by an ROHom-based coefficient (F_ROH_), calculated based on genome-wide homozygosity levels at the generational level. Note that the broken lines in ‘Stevens’, ‘Wilcox’, and ‘Pilgrim’ are due to generations where sequencing data are unavailable. **(B)** Inbreeding levels at the chromosomal level, averaged across generations.

We explored inbreeding levels within the cranberry lines at both the generational and chromosomal levels. Inbreeding tended to increase at the generational level, with lower inbreeding levels in ‘Ben Lear’ overall (**Figure 4A**). The chromosomal F_ROH_​ values shown in **Figure 4B** represent averages across all available generations for each cultivar. Because the four cultivars were represented by different numbers of selfing generations, with ‘Ben Lear’ extending through S10, these averages should be interpreted with this difference in sampling in mind (**Figure 4B**). The inbreeding coefficients were calculated based on the levels of homozygosity throughout the genome and can be found in **Supplementary Table 3**. Higher inbreeding levels are generally associated with reduced genetic diversity correlated with lower genetic diversity and an increased probability of phenotypically expressing deleterious recessive alleles (Charlesworth and Willis, 2009). However, all cultivars tested were remarkably tolerant of inbreeding from a viability standpoint as well as the phenotypic traits measured.

**Figure 5:**
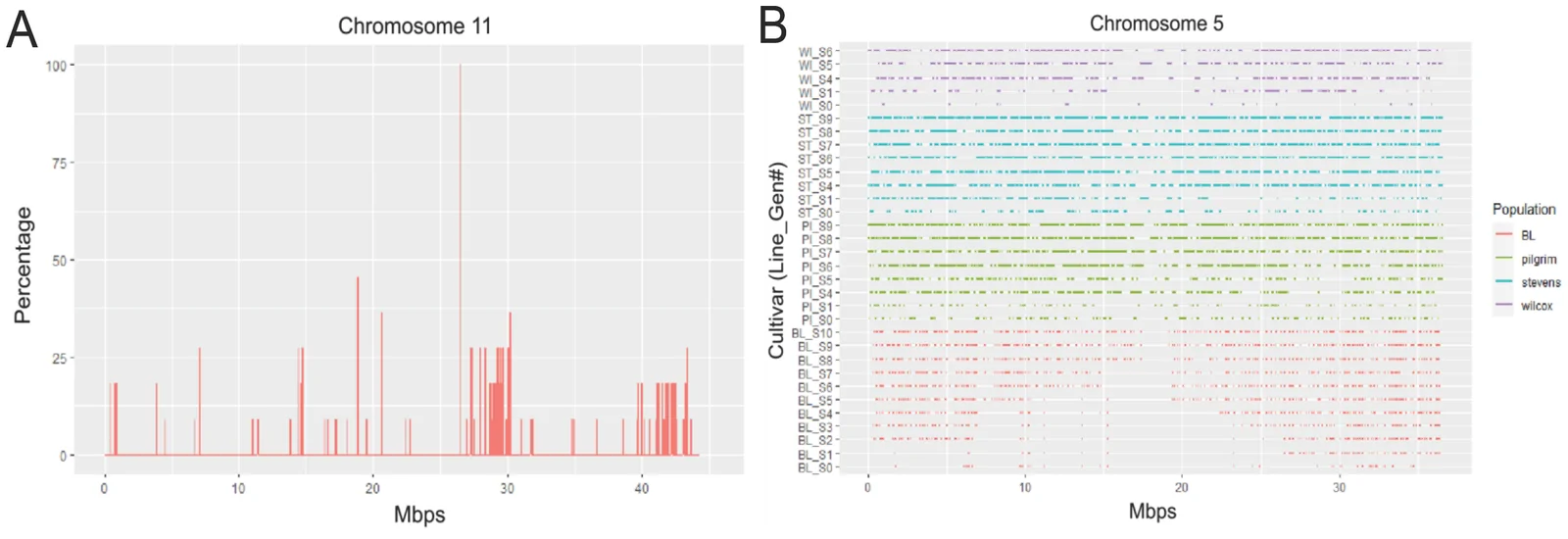
ROHet and ROHom are identified in representative chromosomes. **(A)** SNPs found in ROHet in chromosome 11 across ‘Ben Lear’ generations, expressed by the percentage of generations in which the SNP falls inside a ROHet. Notably, an ROHet is identified in every generation of the ‘Ben Lear’ line at ~26 Mbps. **(B)** ROHom identified in chromosome 5 across generations of the four cultivars. For clarity, enlarged, full-size versions of each panel are provided in the **Supplementary Data**).

The chromosomal distributions of ROHet and ROHom were investigated to identify genomic regions potentially associated with inbreeding tolerance. In particular, the same ROHet was found in every generation of ‘Ben Lear’ in chromosome 11 at ~26 Mbps (**Figure 5A**). We also observed trends across the four cultivars for both ROHet and ROHom. As expected, homozygosity levels increased across successive selfing generations, as illustrated for chromosome 5 (**Figure 5B**). In this chromosome, ROHom in ‘Stevens’, ‘Wilcox’, and ‘Pilgrim’ progressively merged into longer contiguous runs during the early generations and became relatively evenly distributed across the chromosome. In contrast, the ‘Ben Lear’ line exhibited substantially less ROHom merging even by generation S10, with little ROHom across the central portion of chromosome 5. Comparable distributions for all chromosomes are provided in the Graphs directory in the **Supplementary Data**.

Examination of the genome-wide ROHet and ROHom distributions revealed little consistency among the four cultivars. There were also multiple instances where ROHet was present in more recent generations and missing in older generations. These discontinuities may reflect the stringent variant filtering criteria, particularly the minimum read depth and call rate, or the revisions in genotyping availability due to changed site variation during inbreeding and the enzymatic and fragment size selectivity of GBS. Finally, there were many instances where particular generations of a cultivar did not have any ROHet in certain chromosomes. These observations indicated a lack of consistent patterns that could explain inbreeding intolerance in the four lines.

### 2.4 Identification of regions of maintained heterozygosity and exploring these regions with gene annotation

We investigated regions of maintained heterozygosity in the four cultivar lines and explored the annotation of genes in these regions. As shown in **Figure 5A**, the ROHet in chromosome 11 of ‘Ben Lear’ was demonstrated to be a very conserved region of maintained heterozygosity in every generation. We hypothesized that identifying more of these regions can provide biological significance to the observed maintenance of the inbreeding tolerance of ‘Ben Lear’. A region of maintained heterozygosity was defined as one that was present in at least half of the generations of the cultivar. As a result of this analysis, we found 11 such regions in ‘Ben Lear’, three in ‘Stevens’, one in ‘Wilcox’, and one in ‘Pilgrim’ (**Table 1**). All the identified regions were unique to their respective cultivar, as there were no conserved regions of maintained heterozygosity shared among the four cultivars. Notably, the region identified in ‘Pilgrim’ was lost only in the last generation, which could indicate the region is not of critical importance to maintenance of inbreeding tolerance or viability. In ‘Stevens’ and ‘Wilcox’, there were no such regions of maintained heterozygosity that were also lost in the last generation. We identified the genes intersecting each of these regions in **Table 1** as well as their putative functions, according to two annotated genome files for *V. macrocarpon*, one from ‘Ben Lear’ and another from ‘Stevens’. Annotated self-incompatibility genes in the cranberry reference genome did not overlap any regions of maintained heterozygosity from **Table 1** (data not shown).

**Table 1:** Regions of maintained heterozygosity across the four cultivars.

Cultivar	Chrom #	# SNPs	Start (bp)	End (bp)	Length (bp)	# Genes	Putative gene functions
Ben Lear	1	66	18936808	18953093	16286	1	Outer envelope pore protein 37, chloroplastic
Ben Lear	4	13	8355182	8372327	17146	4	Methyltransferase; serine threonine-protein kinase
Ben Lear	5	42	11340466	11362570	22105	1	Cullin-associated NEDD8-dissociated protein 1
Ben Lear	5	55	12101727	12121482	19756	2	Pectin acetylesterase 10; polysaccharide biosynthesis
Ben Lear	5	12	15658408	15673471	15064	1	Hypothetical
Ben Lear	5	48	16495205	16510260	15056	3	Gibberellin receptor; Hypothetical
Ben Lear	5	23	16635241	16650561	15321	2	Protein kinase domain
Ben Lear	5	53	19026440	19043211	16772	2	Membrane-associated protein vipp1, chloroplastic; universal stress protein A-like protein
Ben Lear	11	22	26476118	26503528	27411	8	Annexin repeats; Hypothetical
Ben Lear	12	10	36739269	36754750	15482	1	Formate dehydrogenase (NAD+)
Stevens	1	17	12097249	12130488	33240	6	3-ketoacyl-coa synthase; Hypothetical
Stevens	1	16	41254672	41317189	62518	7	GroES chaperonin family; Plant self-incompatibility protein S1; Wuschel-related homeobox
Stevens	10	9	13152454	13198165	45712	6	Protein PHOSPHATE-INDUCED 1
Wilcox	10	11	20865549	20883635	18087	1	G-type lectin S-receptor-like serine threonine-protein
Pilgrim	12	10	30799079	30880722	81644	6	Cytochrome P450; Alpha-1,4-glucan-protein synthase [UDP-forming]; Myb-related protein; F-box Kelch-repeat protein; E3 ubiquitin-protein ligase

Additionally, associated Gene Ontology (GO) IDs and term names are presented in **Supplementary Table 4**. Some of these genes were not annotated, and their products were labeled as hypothetical proteins, as shown in **Supplementary Table 5**. This shows a critical need for further studies to accurately and comprehensively annotate some of the genes in the cranberry genome. In particular, we identified eight genes within the previously mentioned ROHet in chromosome 11 of ‘Ben Lear’, although many of their functions were hypothetical. These genes represent candidates that may contribute to the maintenance of viability in the ‘Ben Lear’ inbred lines.

To complement the current cranberry annotation, we used BLAST to find homologous genes in *Arabidopsis thaliana*. A few of the gene sequences shared homology with genes in *A. thaliana*, as shown in **Supplementary Table 5**. In ‘Ben Lear’, these functions included chloroplast movement and dsRNA-binding. The only homolog found in ‘Stevens’ encodes for cytochrome c. The only gene found in ‘Wilcox’ has a homolog important for anther development. In ‘Pilgrim’, these functions included lipid transport, vacuolar function, and glutamine amidotransferase. These genes, identified among the four cultivars, some novel and yet to be functionally identified, represent candidate genetic factors that potentially contribute to inbreeding response in cranberries.

### 2.5 Conserved functional domains and sequence motifs within regions of maintained heterozygosity

To further explore the potential biological significance of these regions of maintained heterozygosity, we identified motifs common to these regions and functional domains within the intersecting genes. Using MEME (Bailey and Elkan, 1994), we found GAGA/TCTC repeat motifs in every region (**Supplementary Figure S3**). Using NCBI’s Conserved Domain Database (CDD) (Wang et al., 2023), we were able to identify functional domains in many of the intersecting genes (**Supplementary Table 6**). These domains included RNase H-like and cellulose synthase superfamilies in ‘Ben Lear’, leucine-rich repeats in ‘Stevens’, and lipid-transfer proteins in ‘Pilgrim’.

## 3 Discussion

In this study, we investigated the genomic consequences of repeated selfing in four cranberry cultivars by characterizing genome-wide patterns of runs of homozygosity (ROHom) and runs of heterozygosity (ROHet) across successive generations. We sought to better understand how sequential inbreeding alters genomic diversity, identify genomic regions that maintain heterozygosity despite repeated selfing, and assess whether these genomic changes are accompanied by phenotypic evidence of inbreeding depression. Our results demonstrate progressive increases in homozygosity across generations and several regions of maintained heterozygosity that represent promising candidates for future investigation.

The phenotypic response to inbreeding is known to vary among plant species, genotypes, and the trait being measured. In a study of inbred maize lines, populations differed in their response to inbreeding for traits including yield, ear number, and ear height. This variability was thought to be due to differences in genetic potential for inbreeding depression (Burton et al., 1978). Similarly, in eight cassava (*Manihot esculenta*) families, inbreeding depression levels for key traits such as fresh root yield, fresh foliage yield, and plant height were not uniform and varied among the families (Rojas et al., 2009). In the present study, we measured the effect of inbreeding on several traits in cranberries, including overall plant stature (**Figure 1**, top), leaf area, fruit size, and seed number (**Supplementary Tables 7, 8**). Although the extent of phenotypic change varied among cultivars and traits, these observations are consistent with previous reports that the effects of inbreeding are trait- and genotype-dependent.

Herein, we expanded the publicly available repository of cranberry genotyping data and provide a better understanding of its genetic diversity as it pertains to inbreeding. To do this, we provided a resource of sequencing data from multiple cranberry cultivars and accessions from selfing cycles. This is the first study investigating ROHet and ROHom trends in cranberries, as well as the first to analyze them in the context of consecutive generations of *V. macrocarpon.* Importantly, we also identified several regions of maintained heterozygosity in the four cultivars. The function of the genes within these regions can help identify the biological underpinnings of inbreeding intolerance in cranberries. In **Supplementary Table 5**, several genes with known functions or homologs in ‘Ben Lear’ are chloroplastic, including outer envelope pore protein 37 (OEP37) and membrane-associated protein VIPP1. These genes may be important for chloroplast envelope maintenance, as was demonstrated for VIPP1 in *Arabidopsis thaliana (*Zhang et al., 2012). Other genes include formate dehydrogenase, cullin-associated NEDD8-dissociated protein 1, and pectin acetylesterase. These play roles in plant metabolism and stress responses (Lou et al., 2016), growth and structure (Cheng et al., 2020), and structural regulation (Gou et al., 2012), respectively.

In ‘Stevens’, known genes include cytochrome c with preferential expression in the anther and phosphate-induced-1 (PHI-1) family members. As a crucial mitochondrial protein involved in cellular respiration and energy production, cytochrome c expression in the anther may be linked to the high energy demands of pollen production and germination (Ribichich et al., 2001). PHI-1 genes have been shown to be involved in cellulose biosynthesis regulation in *Arabidopsis thaliana (*Yu et al., 2024).

In ‘Wilcox’, the sole identified gene encodes for a receptor-like protein kinase that has been shown to positively regulate plant response to salt stress (Sun et al., 2013). In ‘Pilgrim’, annotated functions are involved in key cellular processes including metabolism and stress responses, such as cytochrome P450 (Xu et al., 2015) and Myb-related protein (Ambawat et al., 2013). Additionally, other annotated genes mediate ubiquitination of other proteins and regulate development, such as F-box Kelch-repeat protein (Zhang et al., 2013) and E3 ubiquitin-protein ligase (Mazzucotelli et al., 2006). Notably, this region in ‘Pilgrim’ became homozygous in the final generation, suggesting maintenance of heterozygosity in these genes in cranberry is not required for inbreeding tolerance. The above-described genes were all located in regions of maintained heterozygosity in their respective cultivars, indicating their heterozygosity may be important for the viability of the cranberry, supported by the various functions in plant growth, development, and regulation. Investigating these genes further may provide valuable insight and targets for crop breeding. These also represent only a fraction of the total genes that maintain heterozygosity, as the others were still hypothetical with no known homologs or deduced functions.

The effects of inbreeding were evident in the genotypic data presented from selfed cranberry cycles. As expected, we observed increases in inbreeding levels as the cranberry generation number increased, which was directly correlated to increased homozygosity levels across the genome. In a study of *Arabidopsis arenosa* populations, deleterious phenotypes were linked to increased homozygosity and the presence of ROHom (Barragan et al., 2024). It is very possible that an increase in homozygosity and deleterious alleles might confer loss of viability in other cranberry lines. However, despite progressive increases in homozygosity, three of the four cultivars remained viable throughout successive generations of selfing and exhibited relatively modest changes in the phenotypic traits measured. Wilcox represented an exception, as a cyclical translocation limits its viability during continued selfing (Ortiz and Vorsa, 2004). These observations suggest that the cranberry cultivars examined here may be relatively resilient to repeated selfing for the traits evaluated compared to other plant species. For example, the highbush blueberry (*Vaccinium corymbosum*) exhibits low inbreeding tolerance and self-infertility, showing a decrease in seed number and an increase in seed abortion with increasing relatedness among parents (Krebs and Hancock, 1990). In contrast, we did not observe clear trends in leaf or fruit size in cranberries with increasing selfing cycles (**Figure 2**). Analyzing the genome’s architecture may provide important information on the population’s genetic background and potential routes to maintain diversity and fitness in some populations (Peripolli et al., 2017). In cranberries, doing so can help us understand the consequences of inbreeding depression on different cultivars for better breeding program management.

From what we have reported here, there is potential for further investigation to reveal additional insights into inbreeding stability in cranberries. About half of the genes, 24/51 (47%), intersecting the regions of maintained heterozygosity were labeled as hypothetical proteins, with some sharing a homologous gene in *Arabidopsis thaliana*. The genes identified in this study, whether annotated or not, may have an impact on the overall viability of the cranberry. One of the limitations of this study is that we mainly collected data for three phenotypic traits, namely, leaf size, fruit size, and seed count. Further phenotypic data can help provide a better understanding of the inbreeding tolerance. An additional limitation of this study is that SNP positions were determined using a single cranberry reference genome, which may not fully reflect the true marker order across all cultivars due to structural genomic variation. As a result, the number and length of ROHom may be underestimated in some genomic regions. Future studies using cultivar-specific reference genomes may improve the accuracy of SNP positioning and ROHet/Hom estimation.

Common regions of maintained heterozygosity among the four cultivars were difficult to identify. The ‘Ben Lear’ genome may provide insights into genomic regions associated with its relatively modest phenotypic changes during selfing, as this cultivar also exhibited comparatively lower calculated inbreeding coefficients even after 10 generations of selfing. In particular, the maintained heterozygous region on chromosome 11 contains eight annotated genes, including several annexin repeat genes. These genes are known to play roles in stress responses and cellular signaling and thus represent promising candidates for future investigation. However, this region could not be evaluated in the remaining cultivars because we lacked sequencing coverage, likely reflecting differences in sequencing strategy, as the ‘Ben Lear’ reference was generated using whole-genome sequencing while the other cultivars were genotyped using GBS. Consequently, it is difficult to determine whether this maintained ROHet is unique to ‘Ben Lear’ or whether comparable regions exist but were not captured in the reduced-representation GBS datasets. More broadly, apparent differences between ‘Ben Lear’ and other cultivars in the number or extent of maintained heterozygosity regions should not be interpreted as evidence of differential inbreeding tolerance without confirmation using comparable WGS datasets. Nevertheless, the genes in the chromosome 11 region, as well as those in the other regions of maintained heterozygosity, represent intriguing candidates for future studies, including whole-genome sequencing of additional cultivars and experimental validation of these genes to determine their roles in maintaining viability and other horticulturally important traits. These data will improve our understanding of the genetic consequences of inbreeding in cranberries and may provide insights applicable to closely related species, such as blueberries and other small fruit crops.

## 4 Conclusion

This study presents the first long-term inbreeding analysis in cranberry, highlighting cultivar-specific responses and genome-wide patterns of heterozygosity and homozygosity. Regions of maintained heterozygosity represent candidate genomic regions that may be associated with inbreeding response. The genes identified within these regions, including those involved in metabolism, stress response, and cellular regulation, offer potential targets for breeding programs aimed at improving genetic vigor. By expanding public cranberry genomic resources and quantifying ROHet and ROHom trends across selfed generations, this work provides deeper insights into cranberry inbreeding dynamics.

## 5 Materials and methods

### 5.1 Plant growth

In this study, four common cranberry parental cultivars, namely ‘Stevens’, ‘Wilcox’, ‘Pilgrim’, and ‘Ben Lear’ were used. All plants were grown and maintained in a greenhouse in Chatsworth, NJ. The inbred lines were generated over a period of 27 years. Experimental research and field studies on plants (either cultivated or wild) in this study, including the collection of plant material, comply with relevant institutional, national, and international guidelines and legislation.

### 5.2 Plant Crosses

Plants to be used for crosses were allowed to flower in the greenhouse. Flowers were emasculated 1–2 days before anthesis and hand-pollinated 5–7 days later. The first round of inbreeding involved the self-pollination of all cultivars described in this manuscript (i.e., ‘Ben Lear’, ‘Stevens’, ‘Wilcox’, and ‘Pilgrim’). Fruits were allowed to ripen on the vine, and seeds were extracted at the end of the season (September-October). Seeds were stored at 4 °C for the winter and planted in the spring (March-April) in the greenhouse. The seedlings were grown in the greenhouse until flowering (2–3 years after seeds were planted). The selfing cycles were repeated as plants/flowers/fruit became available. At the time these data were collected, plants were up to the 9th selfed generation (S9). Over the years, some plants were lost to disease, etc., so some generations for some of the cultivars are incomplete. It is important to note that each selfing cycle likely purges some deleterious genes from each line. Still, in contrast to highbush blueberry (*V. corymbosum*), selfing in cranberry yields viable progeny even after many cycles. Unfortunately, each cycle would lead to a logarithmic increase in the total number of progenies. As a result, proper care for such large populations would soon become untenable. Thus, we limited each generation for each variety to 3–6 individuals. Those ‘saved’ were randomly chosen (i.e., not selected for vigor).

### 5.3 Plant measurements

Leaves were collected from representative plants of available inbreeding generations and observed under a dissecting scope (Leica MZ16FA, Leica Microsystems, Wetzlar, Germany). Images were captured using a Nikon DS-Fi1c camera (Nikon Instruments Inc., Melville, NY, USA) mounted on the scope. Leaves in the captured images were measured (length and width in mm) using Nikon NIS-Elements-BR software (Nikon Instruments Inc.). Three leaves were measured for each accession. Average size and area were calculated. Note that all leaves were current-year growth and fully expanded. The fruit was fully ripened (i.e., turned red and no longer expanding). On the other hand, fruits were collected when available from the inbreeding generations. Length and width of the fruit were measured with digital calipers in mm. Each fruit was then sliced to collect the seeds. Seeds were counted and weighed on an analytical balance. Average leaf area, average fruit width, and average seed count were calculated for each generation across the representative plants of each cultivar. Phenotypic differences across generations were tested using one-way ANOVA followed by Tukey’s Honestly Significant Difference (HSD) tests. Whole potted plants and whole fruit were photographed with a standard digital camera (Olympus Tough TG-5, Olympus America Inc., Center Valley, PA, USA).

### 5.4 Sequencing

Genotyping by Sequencing (GBS) was performed on each parent and on one randomly selected plant from each selfed generation for each cultivar. DNA was extracted from leaf tissue with a modified CTAB protocol, and GBS libraries were generated and sequenced as in Daverdin et al. (2017) (Daverdin et al., 2017). Briefly, DNA was digested using a dual-enzyme restriction with Pst1 and Nde1 (New England Biolabs Inc., Ipswich, MA, USA). Sequencing of the GBS libraries was performed by Genewiz, Inc. (South Plainfield, NJ, USA) using a 2 × 150 bp output on an Illumina HiSeq. The resulting fastq files were used for downstream genomic analysis steps. For all four cultivars, one fastq file was first demultiplexed using process_radtags from Stacks to retrieve paired fastq files. For ‘Ben Lear’, whole-genome sequencing (WGS) data were also collected. ROHet and ROHom were initially identified using the GBS dataset to maintain consistency with the analyses performed for the other cultivars. Subsequently, the WGS dataset was used for all downstream analyses involving the characterization of ROHet and ROHom in ‘Ben Lear’, owing to its more complete genomic coverage.

### 5.5 Genome alignment and variant calling

The reference cranberry genome used for alignment was sequenced and assembled from the 5th generation self of ‘Ben Lear’ (Kawash et al., 2022), accessed from https://www.vaccinium.org/bio_data/1254430. Paired-end reads from each generation of the four cultivars were aligned to the reference genome using BWA-MEM with default parameters (Li and Durbin, 2009). From the output alignment files, reads were sorted and duplicate reads were marked with samtools (Danecek et al., 2021). Variants were identified using bcftools mpileup and call (Danecek et al., 2021). Variants with a minimum read depth of less than 4 were removed. PLINK was used to convert VCF files to ped/map files and filter out variants with a call rate of less than 90% (Purcell et al., 2007). SNP marker density was visualized using the R package CMplot (Yin et al., 2021) by calculating the number of SNPs with a 1 Mb window size across each chromosome.

### 5.6 ROHom and ROHet identification

The R package detectRUNS (Detectruns: Vignettes/Detectruns.Vignette.Rmd, 2025) was used to identify Runs of Homozygosity (ROHom) and Runs of Heterozygosity (ROHet) using the “consecutive runs” method. To identify ROHoms, the following parameters were used: (1) the minimum number of SNPs was 20; (2) the maximum gap within an ROHom was 1000 kb; (3) the minimum length was 20 kb for ‘Ben Lear’ (because we used WGS data), 25 kb (using GBS data) for ‘Stevens’, ‘Wilcox’, and ‘Pilgrim’; (4) a maximum of one heterozygous SNP was allowed.

To identify ROHets, the following parameters were used: (1) the minimum number of SNPs was 10; (2) the maximum gap within an ROHet was 1000 kb; (3) the minimum length was 15 kb for ‘Ben Lear’ and ‘Wilcox’, 25 kb for ‘Stevens’ and ‘Pilgrim’; (4) a maximum of one homozygous SNP was allowed. The inbreeding coefficients of each generation were also calculated using the formula $F_{ROH}=\frac{\sum \;L_{ROH}}{L_{genome}}$ where $\sum \;L_{ROH}$ is the sum of the length of all ROHom within a cultivar generation, and $L_{genome}$ is the length of the genome.

To identify regions of maintained heterozygosity, a threshold of 0.5 was used so that the regions were present in at least half of the generations of the cultivar. To make graphs of ROHet/ROHom counts and inbreeding levels, matplotlib was used. Graphs of ROHet and ROHom within chromosomes were plotted using detectRUNS built-in functions, plot_Runs(), and plot_SnpsInRuns().

### 5.7 Gene annotation and Gene Ontology (GO) analyses

The regions of maintained heterozygosity were annotated for overlapping genes using bedtools intersect (Quinlan and Hall, 2010) with the gff3 file of the annotated ‘Ben Lear’ genome and using eggNOG-mapper (Cantalapiedra et al., 2021) with the annotated ‘Stevens’ genome. Some of the annotated gene products were hypothetical proteins. The nucleotide sequences of these hypothetical proteins were run through BLAST (Altschul et al., 1990) against the *Arabidopsis thaliana* database at the Arabidopsis Information Resource (TAIR) (Rhee et al., 2003). The GO IDs of the gene products were also used as input to AgBase GORetriever (McCarthy et al., 2006) against the “Plants” database to retrieve GO annotations.

### 5.8 Functional domain and motif identification

The sequences of genes intersecting the regions of maintained heterozygosity were used as input to NCBI’s Conserved Domain Database search tool (Wang et al., 2023). The sequences of the regions of maintained heterozygosity were used as input to MEME (Bailey and Elkan, 1994).

## Data Availability

Raw sequencing data is available here (https://rutgersconnect-my.sharepoint.com/:f:/g/personal/dw749_camden_rutgers_edu/IgBTLFr9IUbiQ5vgaNi7thPbAfhRS_iuCdFgVo8pi_YfwD0?e=2zQJpf) and at NCBI SRA project number PRJNA1370000. For more information, you can reach out to the corresponding author (Iman Dehzangi: i.dehzangi@rutgers.edu).

## Supplementary material

The Supplementary Material for this article can be found online at: Available at: https://rutgersconnect-my.sharepoint.com/:f:/g/personal/dw749_camden_rutgers_edu/IgDuXvaBOzWsT4FWn7FwZ8uvATql7JxlVuLkbqG8xGBe7Rw?e=1eq9LV.
